# Impact of *ASXL1* Gene Alterations on Myelodysplastic Syndrome With Isolated 20q Deletion

**DOI:** 10.1002/cam4.70747

**Published:** 2025-03-06

**Authors:** Yanan Chang, Linlin Liu, Chenghua Cui, Jiange He, Chengwen Li, Yujiao Jia, Ruixue Zhang, Wanyun Wu, Ji Zhou, Jigang Xiao, Zefeng Xu, Tiejun Qin, Qi Sun, Huijun Wang, Zhijian Xiao

**Affiliations:** ^1^ Hematologic Pathology Center Institute of Hematology and Blood Diseases Hospital, Chinese Academy of Medical Sciences & Peking Union Medical College Tianjin China; ^2^ State Key Laboratory of Experimental Hematology Institute of Hematology & Blood Disease Hospital, Chinese Academy of Medical Sciences & Peking Union Medical College Tianjin China; ^3^ Hand and Foot Microsurgery The Second Hospital of Tianjin Medical University Tianjin China; ^4^ Peking University People's Hospital Beijing China; ^5^ MDS and MPN Centre Institute of Hematology and Blood Diseases Hospital, Chinese Academy of Medical Sciences & Peking Union Medical College Tianjin China

**Keywords:** 20q deletion, *ASXL1*, copy number variant, mutation, myelodysplastic syndrome

## Abstract

**Background:**

Isolated 20q deletion [del(20q)] is a recurrent favorable abnormality in myelodysplastic syndrome (MDS) and may cause deletion of the *ASXL1* gene. Meanwhile, *ASXL1* mutations are also common in individuals with MDS. This study aimed to describe the biological and clinical implications of *ASXL1* mutations and deletion in newly diagnosed MDS patients with isolated del(20q).

**Methods:**

Gene mutation and copy number alterations in 178 newly diagnosed MDS patients with isolated del(20q) were analyzed using DNA next generation sequencing.

**Results:**

Twenty‐five (14%) of 178 patients were found to have *ASXL1* mutations, which exhibited lower absolute neutrophil counts (ANC) (*p* = 0.006), a higher percentage of bone marrow blasts (*p* = 0.001), more mutant genes (*p* < 0.001), higher IPSS‐R (*p* = 0.038) and IPSS‐M (*p* = 0.001) risk groups. Furthermore, *ASXL1* mutations were preferentially associated with mutations in *U2AF1*, and most *ASXL1* mutations (68%) were observed as subclonal lesions. *ASXL1* frameshift mutations were associated with a worse prognosis in MDS patients with low blasts (MDS‐LB) (*p* = 0.043), but not in those with increased blasts (MDS‐IB). Twenty‐two (26.8%) of 82 patients were found to have *ASXL1* deletion, which exhibited a lower IPSS‐M risk group, lower platelet counts, higher ANC levels, and higher hemoglobin levels compared to *ASXL1* patients^only‐mut^ and *ASXL1* ^wt^ patients. Two (2.4%) of the 82 patients exhibited biallelic *ASXL1* inactivation (*ASXL1* ^mut&del^).

**Conclusions:**

*ASXL1* mutations are one of the late genetic events in MDS patients with isolated 20q deletion, and different types of *ASXL1* gene alterations have distinct clinical and biological characteristics.

## 1 Introduction

Myelodysplastic syndrome (MDS) represents a heterogeneous group of clonal bone marrow disorders characterized by dysplastic maturation of hematopoietic cells, peripheral cytopenias, and a variable risk of acute myeloid leukemia (AML) [1, 2]. Del(20q) is a recurrent chromosomal abnormality and is observed in approximately 5% of MDS cases and 7% of t‐MDS cases, occurring as either an early or late event [3]. Patients with del(20q) associated with a complex karyotype have a poor or very poor risk, while those with isolated del(20q) have a favorable prognosis, especially when it occurs at initial presentation [3, 4, 5, 6]. Given that the pathogenesis and progression of MDS are driven by multiple cooperating genetic and chromosomal abnormalities [7, 8, 9, 10, 11], the potential role of isolated del(20q) as a hallmark of a relatively homogeneous MDS subtype warrants careful evaluation.

*ASXL1*, located at 20q11.21, can be deleted in approximately 20% to 50% of patients with del(20q) and can be detected using single nucleotide polymorphism arrays (SNP‐A), array comparative genomic hybridization (aCGH), or fluorescence in situ hybridization (FISH) [12, 13, 14, 15]. *ASXL1* mutations are present in 15% to 22% of MDS patients [16, 17], and are more common in MDS with intermediate or high‐risk cytogenetic patterns [18, 19]. Additionally, *ASXL1* mutations can serve as independent risk factors, associated with decreased overall survival (OS) and shorter time to progression to AML [20, 21]. However, the incidence, biological and clinical features of *ASXL1* mutations and deletion in newly diagnosed MDS with isolated del(20q) are less clear.

In this study, we analyzed the mutational landscape and copy number variations (CNVs) in a cohort of MDS patients with isolated del(20q) and examined the genotype–phenotype associations of *ASXL1* alterations and their impact on survival.

## 2 Materials and Methods

### 2.1 Study Design and Patient Selection

The cohort comprised 178 consecutive, newly diagnosed patients with primary MDS and isolated del(20q) (Figure S1). Deletion of 20q was the only cytogenetic aberration, defined as the presence of ≥ 2 metaphases with isolated del(20q) detected through conventional chromosome banding of bone marrow metaphase samples. All patients were required to undergo bone marrow examination, as well as cytogenetic and molecular evaluations at diagnosis. All patients were diagnosed according to the 2016 revised criteria of the World Health Organization (WHO2016) [22] and classified using the Revised International Prognostic Scoring System (IPSS‐R) [23] and molecular IPSS (IPSS‐M) [24]. Patient characteristics are summarized in Table S1. Treatment data were available for 161 (90.4%) patients. A total of 74 (41.6%) patients received immunosuppressive drugs, including cyclosporine and thalidomide. Thirty‐two (18%) patients received hypomethylating agents with or without venetoclax, 30 (16.9%) received erythropoietin with or without transfusions, 16 (9%) underwent allogeneic hematopoietic stem cell transplantation, and 9 (5%) received other treatment. Treatment response was assessed based on criteria established by the MDS International Working Group [25, 26]. Follow‐up data were available for 159 (89.3%) patients. Patient survival was calculated from the date of diagnosis to either death or loss to follow‐up, or censored at stem cell transplantation. The median follow‐up time was 21 months (interquartile range [IQR], 11–40 months). All cases included in this study received approval from the ethics committees of the Chinese Academy of Medical Sciences and Blood Disease Hospital, and informed consent was obtained from patients in accordance with the Declaration of Helsinki.

### 2.2 Conventional Chromosomal Analysis

Conventional chromosomal analysis was conducted on G‐banded metaphase cells prepared from unstimulated 24‐h bone marrow aspirate cultures. Twenty metaphases were analyzed, and results were recorded in accordance with the International System for Human Cytogenetic Nomenclature (ISCN) 2020 [27].

### 2.3 DNA Next‐Generation Sequencing

Genomic DNA was extracted from bone marrow mononuclear cells following the manufacturer's instructions (TIANGEN, China). Out of 178 samples processed, 96 were detected by DNA next‐generation sequencing (NGS) targeted panel, whereas 82 were detected by a dual‐approach DNA NGS platform which synergistically integrates DNA NGS targeted panel analysis with SNP probe. A targeted NGS panel covering 267 frequently mutated genes in hematological malignancies (Table S2) was applied to detect gene mutations and subjected to massive parallel sequencing using the Illumina NovaSeq 6000. The average gene coverage was 98%, and the average read depth was 2000×. DNA sequencing data were aligned to the GRCh37/hg19 reference genome, and single nucleotide variants (SNVs) and insertions/deletions (Indels) were annotated and filtered using various databases, including COSMIC, ClinVar, HGMD, ExAC, ESP6500, GnomAD, and dbSNP, etc. Remaining variants were manually reviewed and classified according to the AMP/ASCO/CAP Standards and Guidelines for Somatic Variant Interpretation and Reporting [28, 29, 30]. Genome‐wide SNP profiling contains more than 1 million polymorphic SNP markers distributed across the genome. The SNP data were algorithmically fused with NGS results through our proprietary FusionView bioinformatics pipeline. Loss of heterozygosity (LOH) detection was systematically implemented using SNP ‐derived B‐allele frequency data processed through our proprietary bioinformatics pipeline. CNV and LOH analyses were conducted based on normalized read counts and SNP distribution patterns.

### 2.4 Ancestral vs. Sub‐Clonal Variants

To analyze ancestral and subclonal events in *ASXL1*‐mutated patients, copy number‐adjusted variant allele fractions (VAFs) were used to estimate the clonal hierarchy of each sample harboring two or more mutations [31]. Mutations with the highest VAF were defined as ancestral/dominant mutations indicating ancestral origin, while those with similar VAFs (differences of less than 5%) were classified as co‐dominant [32, 33, 34].

### 2.5 Statistical Analysis

Fisher's exact test were used to compare categorical variables, while the Mann–Whitney U test or Kruskal–Wallis H test was employed for continuous variables. All statistical analyses were conducted using SPSS version 24.0 (IBM, Armonk, NY, USA) and R software. Correlations between mutations were assessed using Spearman coefficients and *p*‐values were adjusted by Bonferroni's correction. OS was measured from the time of diagnosis to the last follow‐up or death and was censored at the time of hematopoietic stem cell transplantation. Survival curves were plotted using the Kaplan–Meier method and analyzed with the log‐rank test. All *p*‐values were two‐tailed, and *p* < 0.05 was considered statistically significant.

## 3 Results

### 3.1 Spectrum of Gene Mutations and Copy Number Changes in the del(20q) Cohort

**FIGURE 1:**
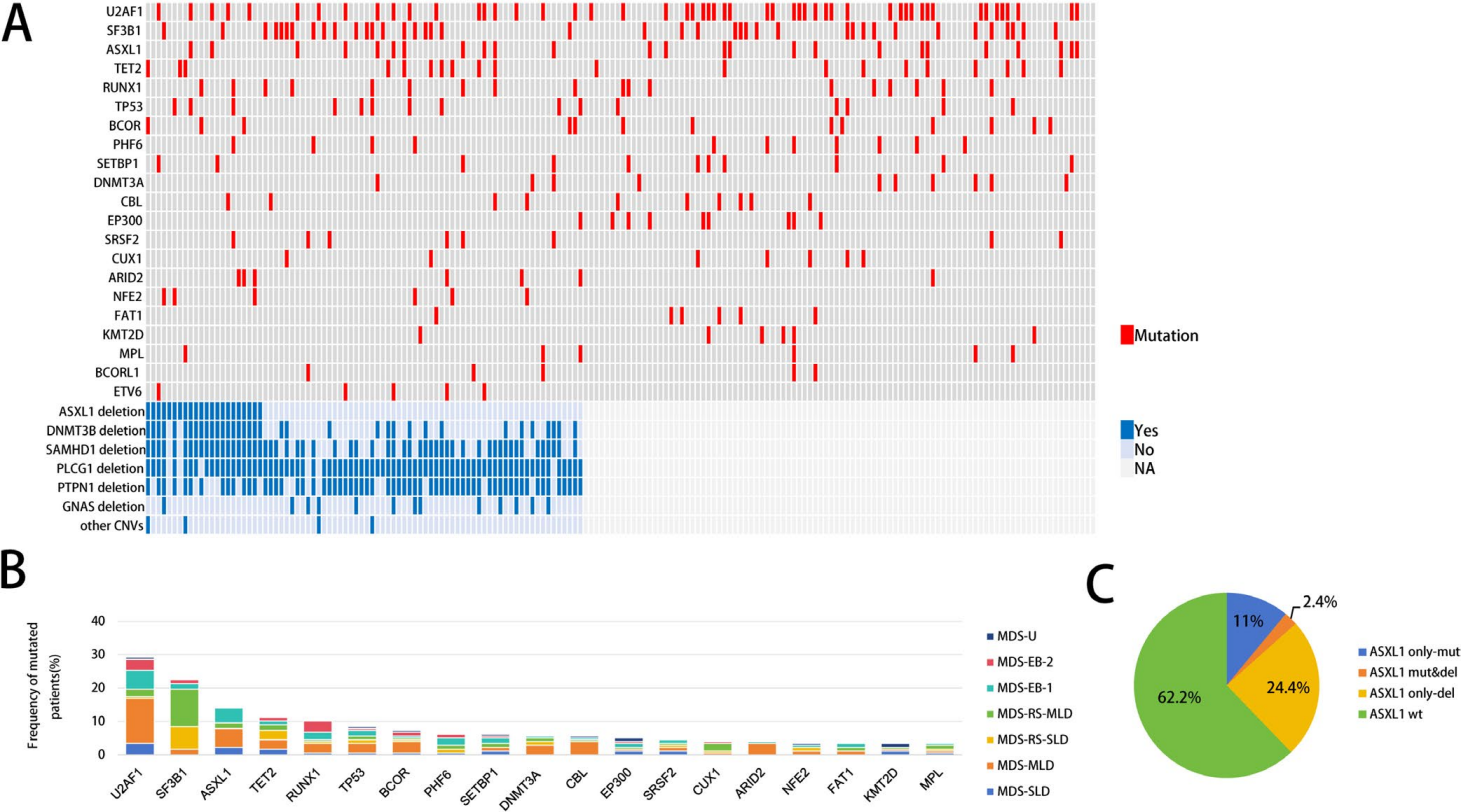
Genetic landscape of newly diagnosed MDS with isolated del(20q). (A) Mutations with a frequency greater than 3% in 178 MDS cases with isolated del(20q) are shown at the top. Genes with microdeletions on chromosome 20q and CNVs on other chromosomes in 82 MDS cases with isolated del(20q) are shown. Each column represents one patient, and each row corresponds to a gene. Deleted cases are shown in dark blue, and mutated cases in red. NA, Not Available. (B) Frequency of 19 significantly mutated genes (> 3%) across different WHO subtypes, shown in different colors. (C) Pie chart showing the distribution of patients with *ASXL1* mutations and/or deletion among 82 MDS cases with isolated del(20q).

Targeted panel NGS was performed on 178 patients. Among the 178 patients, 165 (92.7%) had at least one gene mutation: 34 (19.1%) with one mutation, 46 (25.8%) with two mutations, 35 (19.6%) with three mutations, and 50 (28.6%) with more than three mutations. Twelve genes were mutated in more than 5% of patients, including *U2AF1* (29.2%, 52/178), *SF3B1* (22.5%, 40/178), *ASXL1* (14%, 25/178), *TET2* (11.2%, 20/178), *RUNX1* (10.1%, 18/178), *TP53* (8.4%, 15/178), *BCOR* (7.3%, 13/178), *PHF6* (6.2%, 11/178), *SETBP1* (6.2%, 11/178), *DNMT3A* (5.6%, 10/178), *CBL* (5.6%, 10/178), and *EP300* (5.1%, 9/178). The overall distribution of gene mutations (> 3%) is shown in Figure 1A,B. Among the 178 patients, 82 underwent CNV analysis. Genes with microdeletions on chromosome 20q include *ASXL1* (20q11.2), *DNMT3B* (20q11.21), *SAMHD1* (20q11.23), *PLCG1* (20q12), *PTPN1* (20q13.13), and *GNAS* (20q13.32) (Figure 1A). Additionally, four cases exhibited CNVs on chromosomes other than chromosome 20 (median size: 73 kb, range 43–799 kb)，which were subcytogenetic‐level alterations undetectable by conventional karyotyping (Table S3).

Combined analysis of *ASXL1* deletion and mutations in 82 patients revealed the following findings: *ASXL1* mutations^only‐del^ were found in 20 (24.4%) patients, *ASXL1* deletions^only‐mut^ in 9 (11%) patients, *ASXL1* ^wt^ (*ASXL1* ^non‐del&non‐mut^) in 51 (62.2%) patients, and biallelic *ASXL1* inactivation (*ASXL1* ^del&mut^) in two (2.4%) patients (Figure 1C). Among the 25 patients with *ASXL1* mutations, 16 (64%) patients harbored frameshift mutations, 7 (28%) nonsense mutations, and 2 (8%) missense mutations (Table S4).

### 3.2 Clinical Characteristics of Patients With *ASXL1* Mutations

Compared to *ASXL1* non‐mutated patients, *ASXL1*‐mutated patients had a higher proportion of males (84%, *p* = 0.015, Table S1), lower absolute neutrophil count (ANC) levels (median, 0.84 × 10^9^/L vs. 1.49 × 10^9^/L, *p* = 0.006, Figure S2A), more bone marrow blasts (median, 1.5% vs. 0.5%, *p* < 0.001, Figure S2B), and more mutant genes (median, 4 vs. 2, *p* < 0.001, Figure S2C), as well as a higher IPSS‐M risk group (*p* = 0.001) (Figure S2E) and a higher IPSS‐R risk group (*p* = 0.038). According to the WHO 2016 diagnostic criteria, *ASXL1*‐mutated patients had a higher percentage of MDS with the excess blasts subtype (MDS‐EB‐1 and MDS‐EB‐2) compared to *ASXL1* non‐mutated patients (*p* = 0.042) (Figure S2D). No significant differences in age, hemoglobin, or PLT count were observed between the two groups (Table S1).

Focusing on the 142 lower‐risk patients (IPSS‐R score ≤ 3.5), *ASXL1*‐mutated patients exhibited a higher percentage of bone marrow blasts (*p* = 0.002), a greater number of mutant genes (*p* < 0.001), a higher percentage of MDS with excess blasts subtype (*p* = 0.037), and a higher IPSS‐M risk group (p < 0.001) (Table S5). In contrast, among the thirty‐six higher‐risk patients (20.2%, IPSS‐R score > 3.5), *ASXL1*‐mutated patients exhibited only a greater number of mutant genes (*p* = 0.011) compared to *ASXL1* non‐mutated patients (Table S6).

### 3.3 Clinical Features of Patients With *ASXL1* Deletion and Comparison With *ASXL1* Mutations

**FIGURE 2:**
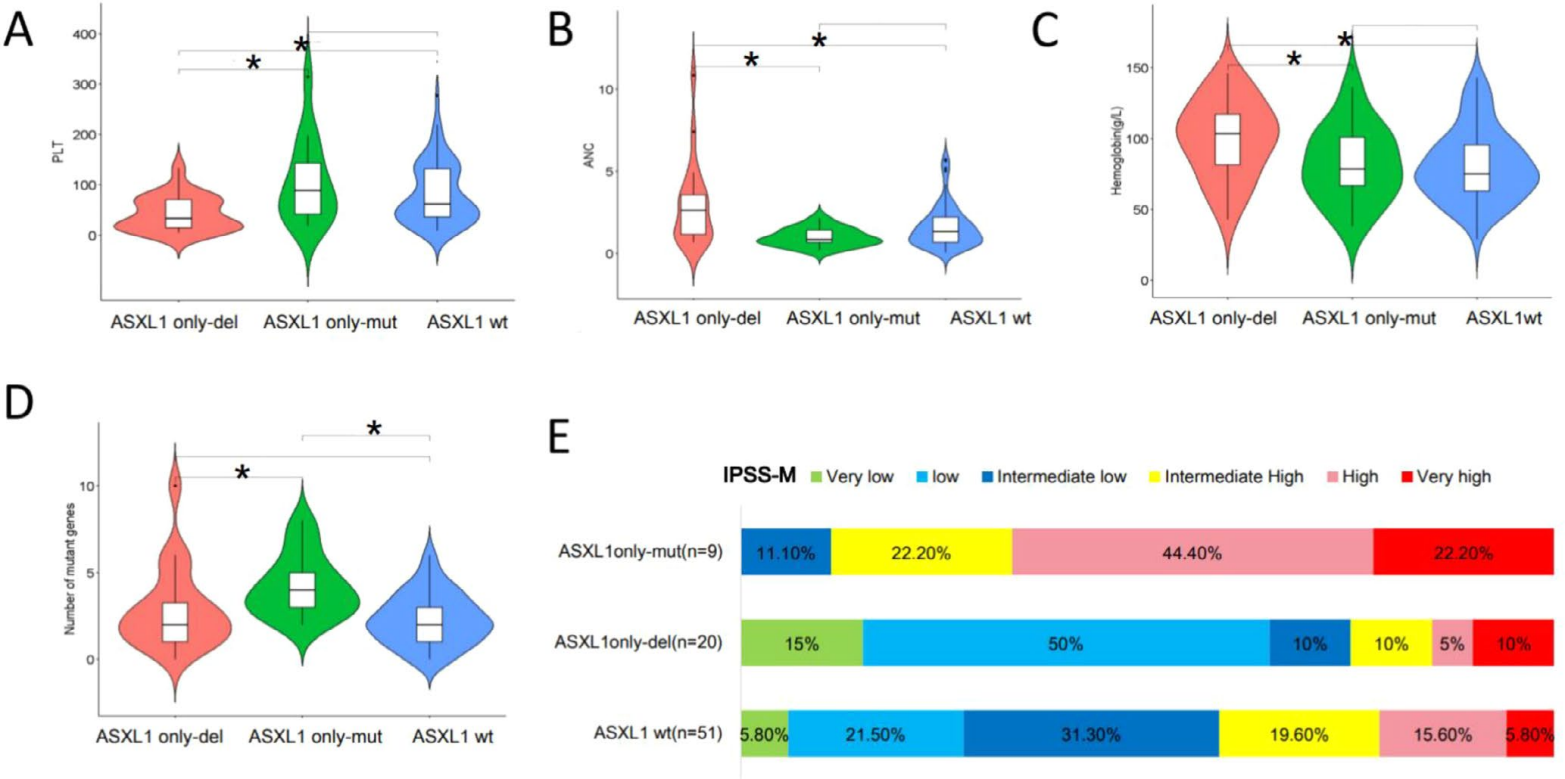
Clinical characteristics comparing the *ASXL1* ^only‐del^, *ASXL1* ^only‐mut^, and *ASXL1* ^wt^ groups. Distribution comparisons of PLT (×10^9^/L) (A), ANC (×10^9^/L) (B), HB (g/L) (C), number of mutant genes (D), and IPSS‐M risk group (E) among *ASXL1* ^only‐del^, *ASXL1* ^only‐mut^, and *ASXL1* ^wt^ groups. *p*‐values are derived from the Wilcoxon rank‐sum test. *p < 0.05.

Patients with *ASXL1* ^only‐del^ had significantly lower platelet counts (median, 33 × 10^9^/L vs 93 × 10^9^/L, *p* = 0.008; 33 × 10^9^/L vs 66 × 10^9^/L, *p* = 0.002; Figure 2A), higher ANC levels (median,2.6 × 10^9^/L vs 0.8 × 10^9^/L, *p* = 0.03; 2.6 × 10^9^/L vs 1.3 × 10^9^/L, *p* = 0.006; Figure 2B), and higher hemoglobin levels (median,103.5 g/L vs 71 g/L, *p* = 0.036; 103.5 g/L vs 74 g/L, *p* = 0.004;Figure 2C), lower IPSS‐M risk group (*p* = 0.002; *p* = 0.032)(Figure 2E) compared to *ASXL1* ^only‐mut^ and *ASXL1* ^wt^ patients. Additionally, patients with *ASXL1* ^only‐mut^ had more mutant genes than those with *ASXL1* ^only‐del^ (median, 4 vs. 2, *p* = 0.017) and *ASXL1* ^wt^ patients (median, 4 vs. 2, *p* = 0.001) (Figure 2D). No significant differences in sex, age, bone marrow blasts, WHO 2016 subtypes, or IPSS‐R risk groups were observed among the three groups (Table S7).

### 3.4 Clonal Architecture and Dynamics of *ASXL1* Mutations

**FIGURE 3:**
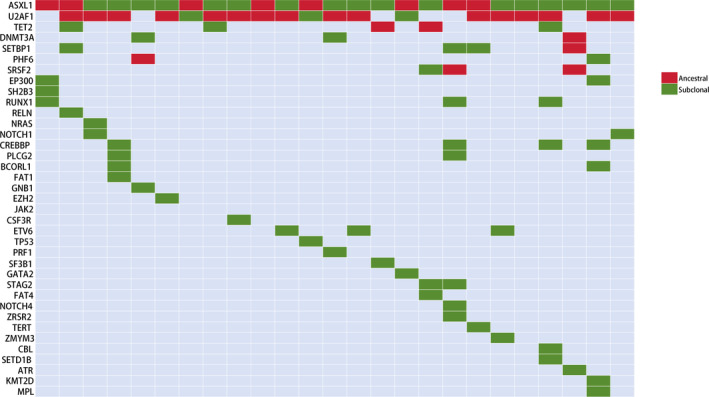
Ancestral and subclonal events in patients with *ASXL1* mutations. Each column represents a patient, and each row corresponds to a gene.

Using copy number‐adjusted VAF, we reconstructed the clonal architecture of *ASXL1*‐mutant patients to determine whether the *ASXL1* mutations were ancestral or subclonal. As shown in Figure 3, all twenty‐five *ASXL1*‐mutant patients carried 1 to 8 other mutations, aside from the *ASXL1* mutations. The *ASXL1* mutation was subclonal in 17 (68%) patients and ancestral in 8 (32%) patients. Ancestral events involving the *ASXL1* mutations were typically accompanied by *U2AF1* co‐mutations (3/8) or subclonal *U2AF1* mutations (3/8).

Six *ASXL1*‐mutant patients with available serial bone marrow samples were enrolled to assess the clonal dynamics of the *ASXL1* mutations (Figure S3). All three MDS‐EB‐1 patients showed clearance of the *ASXL1* mutations after at least three cycles of decitabine or azacitidine (AZA) ± venetoclax (VEN). Three MDS patients (two with MDS‐RS‐MLD and one with MDS‐SLD) who were treated with immunomodulatory therapy and erythropoiesis‐stimulating agents (ESAs) did not exhibit clearance of the *ASXL1* mutations. the frequency of del(20Q) also changed with treatment and tended to be similar to the dynamics of *ASXL1* mutations. Additionally, twenty‐eight *ASXL1* non‐mutant patients with available serial bone marrow samples were also enrolled. Notably, only one (3.6%) *ASXL1* non‐mutant patient developed emerging *ASXL1* mutations during follow‐up.

### 3.5 Correlation Between Genetic Mutations

**FIGURE 4:**
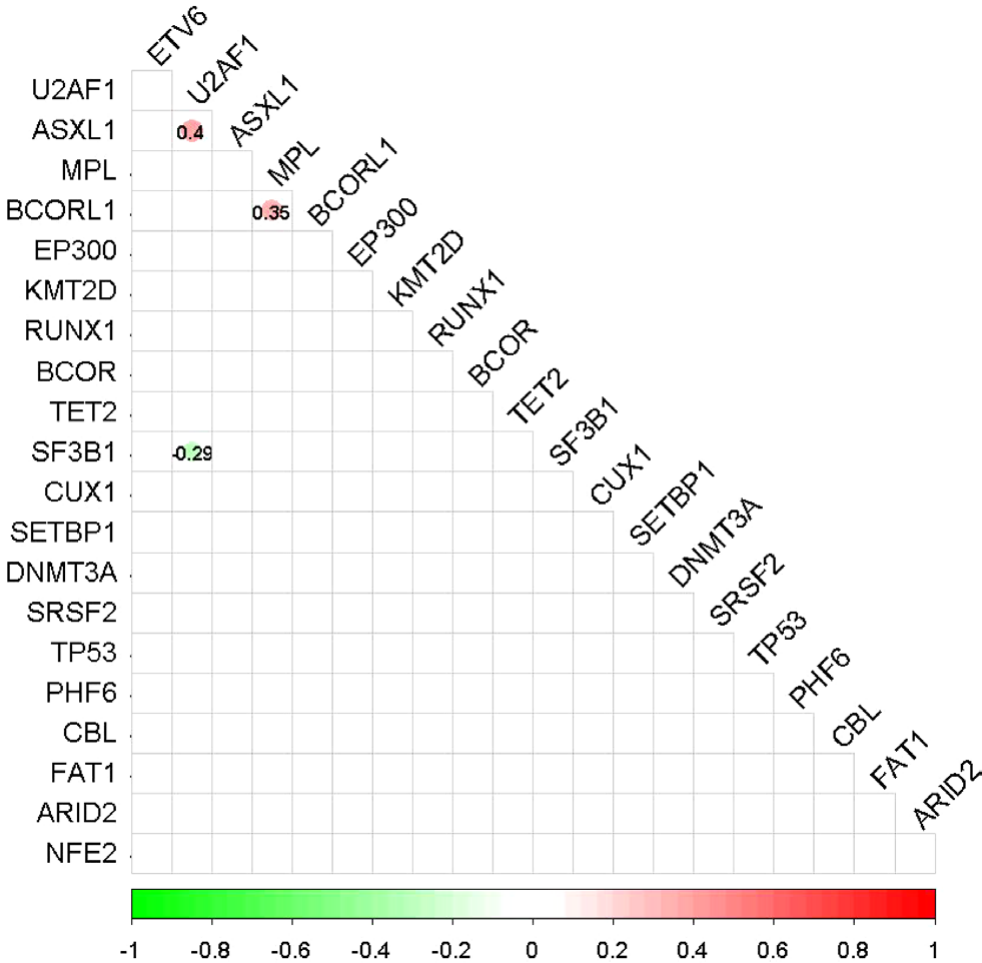
Correlations among the top 21 driver mutations in 178 newly diagnosed MDS cases with isolated del(20q). Comparison of mutation loads among major gene targets in MDS. Statistically significant co‐occurrences and mutually exclusive alterations are shown in red and green, respectively, with correlation rates indicated by values and color gradients. Correlation rates were calculated pairwise. *p*‐values were adjusted by Bonferroni's correction.

The correlations between mutant genes (> 3%) in the cohort are shown in Figure 4. *ASXL1* mutations co‐occurred with *U2AF1* (*r* = 0.4, *p* < 0.0001). Similarly, a co‐mutation was observed between *MPL* and *BCORL1* (*r* = 0.35, *p* < 0.0001). In contrast, *U2AF1* and *SF3B1* mutations were mutually exclusive (*r* = −0.29, *p* < 0.0001).

### 3.6 Survival Analysis

**FIGURE 5:**
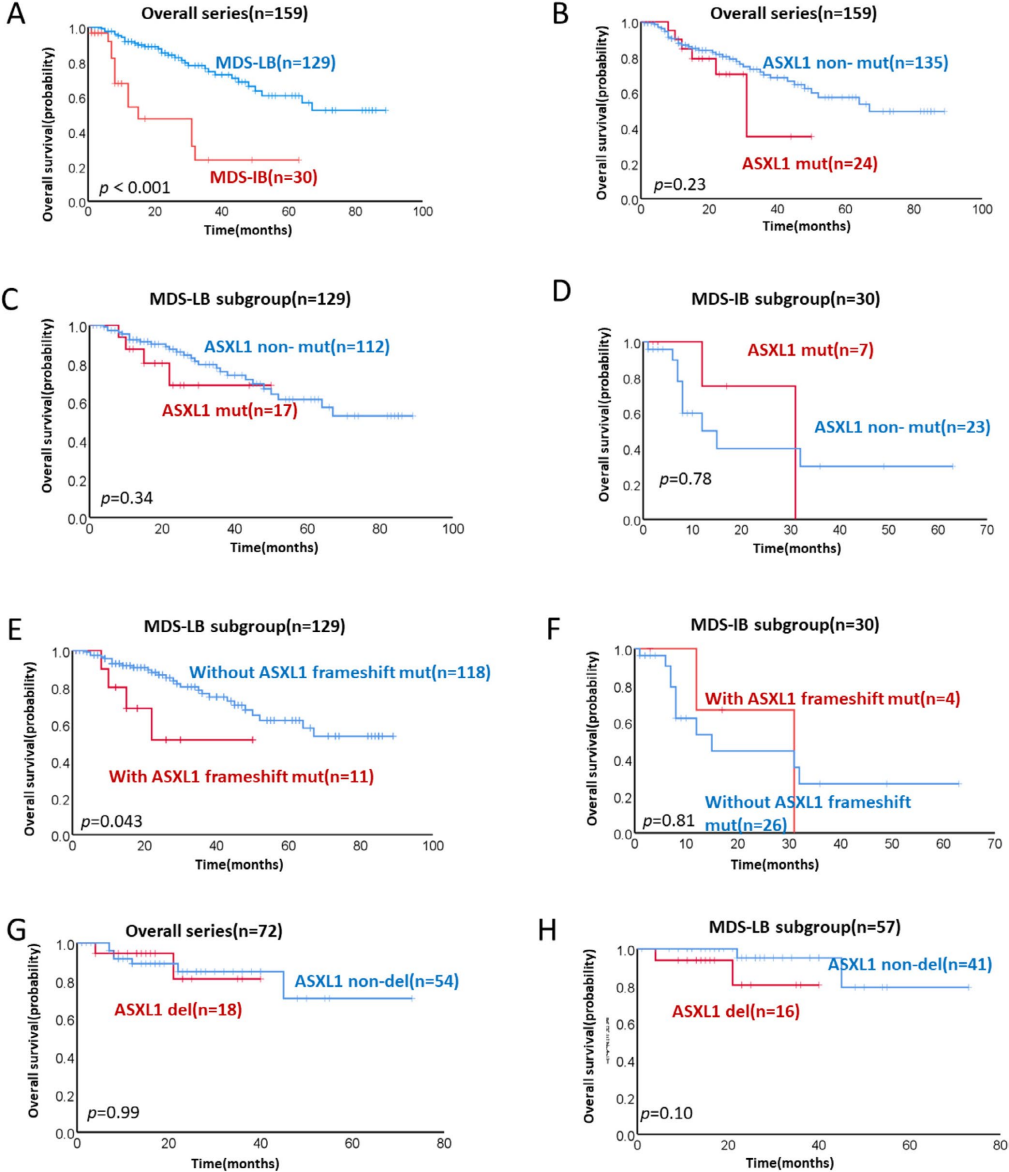
Overall survival in MDS patients with isolated 20q deletion. (A) Survival curves of patients with MDS‐LB and MDS‐IB in the cohort. Survival curves of patients with *ASXL1* mutations in the cohort (B), in MDS‐LB subgroup (C), and in MDS‐IB subgroup (D). Survival curves of patients with *ASXL1* frameshift mutations in MDS‐LB subgroup (E), and in MDS‐IB subgroup (F). Survival curves of patients with *ASXL1* deletion in the cohort (G) and in MDS‐LB subgroup (H). MDS‐LB, MDS with low blasts; MDS‐IB, MDS with increased blasts.

In our cohort, there were 145 (81.5%) MDS patients with low blasts(MDS‐LB), and 33 (18.5%) MDS patients with increased blasts (MDS‐IB), according to the WHO‐2022 classification [35]. Patients with MDS‐IB had a reduced median overall survival (OS) compared to those with MDS‐LB (median OS, 15 months vs. not reached, *p* < 0.001; Figure 5A). Compared to patients without *ASXL1* mutations, *ASXL1* ^mut^ patients tended to be associated with worse OS in the cohort (median OS, 31 vs. 67 months, *p* = 0.23; Figure 5B) and in the MDS‐LB subgroup (median OS, not reached, *p* = 0.34; Figure 5C), but not in the MDS‐IB subgroup (median OS, 31 vs. 12 months, *p* = 0.78; Figure 5D). Considering that *ASXL1* had different mutation types, we further analyzed whether the subtype of *ASXL1* mutations impacts OS in MDS‐LB and MDS‐EB patients, respectively. Compared with patients without *ASXL1* frameshift mutations, patients with *ASXL1* frameshift mutations had a worse prognosis in MDS‐LB patients (median OS, not reached, *p* = 0.043; Figure 5E), but not in MDS‐IB patients (median OS, 31 vs. 15 months, *p* = 0.81; Figure 5F).

Compared to patients without *ASXL1* deletion, patients with *ASXL1* deletion tend to be associated with worse OS in the MDS‐LB subgroup (median OS, not reached, *p* = 0.10; Figure 5H), but not in the cohort (median OS, not reached, *p* = 0.99; Figure 5G).

## 4 Discussion

Our study represents a large cohort investigating genomic alterations by NGS in Chinese patients with MDS harboring isolated del(20q), excluding the effects of anti‐cancer therapy or cytotoxic therapies [36] and other cytogenetic abnormalities [12]. We primarily focused on the alterations of the *ASXL1* gene to investigate whether ASXL1 biallelic inactivation would occur in MDS with isolated del(20q) and to elucidate its unique biological and prognostic characteristics in the patient cohort.

In our study, CNV and mutations were analyzed using NGS. Due to the cost of testing, non‐tumoral tissue controls, for example, oral epithelial cells from every patient, were not collected as matched control samples to ascertain possible germline mutations. We found that *ASXL1* deletion (26.8%) was slightly more prevalent than *ASXL1* mutations (13.4%) in this cohort subset, consistent with prior literature [37] that used FISH and gene sequencing in MDS with isolated del(20q). Notably, two cases (one with MDS‐SLD and one with MDS‐MLD) exhibited both *ASXL1* deletion and mutations in the remaining *ASXL1* gene, indicating biallelic *ASXL1* inactivation, which appears to be a rare occurrence in MDS with isolated del(20q). Bacher et al. [12] also reported that two out of thirty cases exhibited biallelic *ASXL1* inactivation in MDS patients with del(20q), identified through aCGH and gene sequencing. Therefore, the clinical characteristics of patients with both *ASXL1* deletion and mutations should be investigated in future studies involving a larger patient population.

Furthermore, we observed significant differences in laboratory data, including PLT, ANC, hemoglobin, and the number of mutant genes between patients with *ASXL1* deletion and mutations. Several studies have shown that *ASXL1* mutations were more frequently found in males, older patients, and those with lower platelet or hemoglobin levels, predicting an adverse prognostic outcome [37, 38, 39]. Our study demonstrated that *ASXL1* mutations were associated with adverse clinical features, including a higher number of bone marrow blasts and lower ANC levels. While MDS with isolated del(20q) are generally associated with a favorable prognosis, *ASXL1*‐mutated MDS with isolated del(20q) exhibited a higher risk according to IPSS‐R and IPSS‐M, compared to *ASXL1* non‐mutated patients. Notably, in patients with lower risk (IPSS‐R score ≤ 3.5), those with *ASXL1* mutations were still associated with higher risk according to IPSS‐M, while this was not the case for patients with higher risk (IPSS‐R score > 3.5). These results were consistent with those of Jain et al. [40], who demonstrated that *ASXL1* somatic mutations were associated with progression to higher‐risk MDS. Additionally, we found that patients with *ASXL1* ^only‐mut^ were in a higher‐risk IPSS‐M group compared to those with *ASXL1* ^only‐del^. Furthermore, *ASXL1* ^only‐del^ patients had lower PLT levels and higher ANC and hemoglobin levels than those with *ASXL1* ^only‐mut^ or *ASXL1* ^wt^, indicating distinct pathogenic mechanisms for *ASXL1* deletion and mutations in this cohort. In our study, acquired *ASXL1* mutations were the third most common gene mutations in MDS with isolated del(20q), consistent with previous reports [31, 41]. Notably, *ASXL1* remains one of the most frequently mutated genes in patients with isolated del(20q) in bone marrow, even in the absence of morphologic evidence of a myeloid neoplasm; however, it was not associated with disease progression [42].

When multiple driver genes are affected, functional interactions occur between these mutations, influencing both positive and negative selection processes [7, 21]. Therefore, we investigated the correlation among multiple mutations in the cohort of MDS with isolated del(20q). We observed that *ASXL1* mutations co‐occurred with *U2AF1* mutations, while *ASXL1* and *SF3B1* mutations were mutually exclusive, consistent with published data [7, 21, 37, 41, 43, 44]. Secondly, we found that *ASXL1* mutations typically follow other ancestral mutations, particularly *U2AF1*, occurring as a subclonal event in 68% of cases. Less frequently, *ASXL1* mutations represented an ancestral lesion or coexist with *U2AF1* mutations. *U2AF1* mutations may precede the occurrence of *ASXL1* mutations, as observed in 13 out of 24 cases. Thirdly, if *ASXL1* is not mutant at initial diagnosis, it is unlikely to emerge as a secondary or tertiary mutations during follow‐up, as only one out of 28 cases has shown this. However, further analysis of a larger cohort of patients with *ASXL1* mutations is necessary to confirm our findings. In addition, whether the del(20q) is an ancestral event and how the timing may be related to asxl1 and u2af1 mutations is also a topic worthy of study in the future. While our current dataset provides valuable clonal architecture snapshots, we acknowledge the limitations in precisely resolving temporal relationships through bulk sequencing alone. Standard NGS cannot conclusively determine mutation chronology in static samples. Longitudinal single‐cell sequencing or phylogenetic modeling would be required to definitively establish del(20q) as an ancestral event.

Next, we examine the survival impact of *ASXL1* deletion and mutations in the cohort of MDS with isolated del(20q). *ASXL1* deletion was generally associated with a negative clinical outcome in MDS with del(20q) [15, 37]. Martın et al. [37] identified that patients with altered *ASXL1*, either through chromosomal deletion or somatic mutations (*ASXL1* ^del^/ *ASXL1* ^mut^), had lower OS compared to patients with *ASXL1* ^wt^ in isolated del(20q) MDS (median OS: 25 vs. 65 months, *p* = 0.009). Additionally, they noted that patients with *ASXL1* ^only‐mut^ exhibited slightly shorter OS than those with *ASXL1* ^only‐del^ in the cohort of MDS with isolated del(20q), which did not exclude other chromosomal abnormalities (median OS: 11 vs. 15 vs. 36 months for *ASXL1* ^mut^, *ASXL1* ^del^ and *ASXL1* ^non‐del/non‐mut^, respectively; *p* = 0.013). In our study, patients with *ASXL1* ^del^ and/or *ASXL1* ^mut^ exhibited a trend toward worse OS in MDS‐LB; however, since the median OS was not reached, this result did not achieve statistical significance. Additionally, patients with *ASXL1* frameshift mutations had a worse prognosis in MDS‐LB patients (*p* = 0.043), indicating the impact of *ASXL1* mutation types on prognostic evaluation in MDS [45]. As IPSS‐M assigns a fixed risk weight to ASXL1 mutations regardless of variant type, we think a refined risk model considering the different variant types of each mutation may be worth studying. Definitive conclusions from the subset analysis are limited by the relatively short survival follow‐up and the small number of cases.

In summary, this study provides evidence that *ASXL1* deletion and somatic mutation result in different clinical outcomes in patients with MDS and isolated del(20q). This suggests that MDS with isolated del(20q) is a subtype characterized by genetic and clinical heterogeneity, rather than a homogeneous entity. Further evaluation of molecular alterations is needed to assess their impact on clinical outcomes within the favorable‐risk subgroup of MDS patients with isolated del(20q).

## Author Contributions

Z.X. was responsible for designing the study, as well as the modification and approval of the literature. Y.C., L.L., C.C., Z.X., and T.Q. were responsible for collecting and interpreting the patients' data, performing statistical analysis, writing the paper. C.L., Y.J. and R.Z. contributed to interpret the patients' data and providing feedback on the report. J.H. contributed to the statistical process and drawing charts. W.W. and J.Z. contributed to collecting the data. H.W., Q.S., and J.X. provided feedback on the report. All authors reviewed the typescript, approved this version, and agreed to submit it for publication.

## Ethics Statement

All cases included in this study received approval from the ethics committees of the Chinese Academy of Medical Sciences and Blood Disease Hospital.

## Conflicts of Interest

The authors declare no conflicts of interest.

## Supporting information


Data S1.

## Data Availability

The data that supports the findings of this study are available in the Supporting Information of this article.
