# Supplementary material for: Impact of ASXL1 Gene Alterations on Myelodysplastic Syndrome With Isolated 20q Deletion
Source: Cancer Med. 2025 Mar 6;14(5):e70747. doi: 10.1002/cam4.70747 (PMC11883421; doi:10.1002/cam4.70747)
Supplement: Supplementary file 1 — Data S1. [file CAM4-14-e70747-s001.docx]

**Supplemental figure:**

**
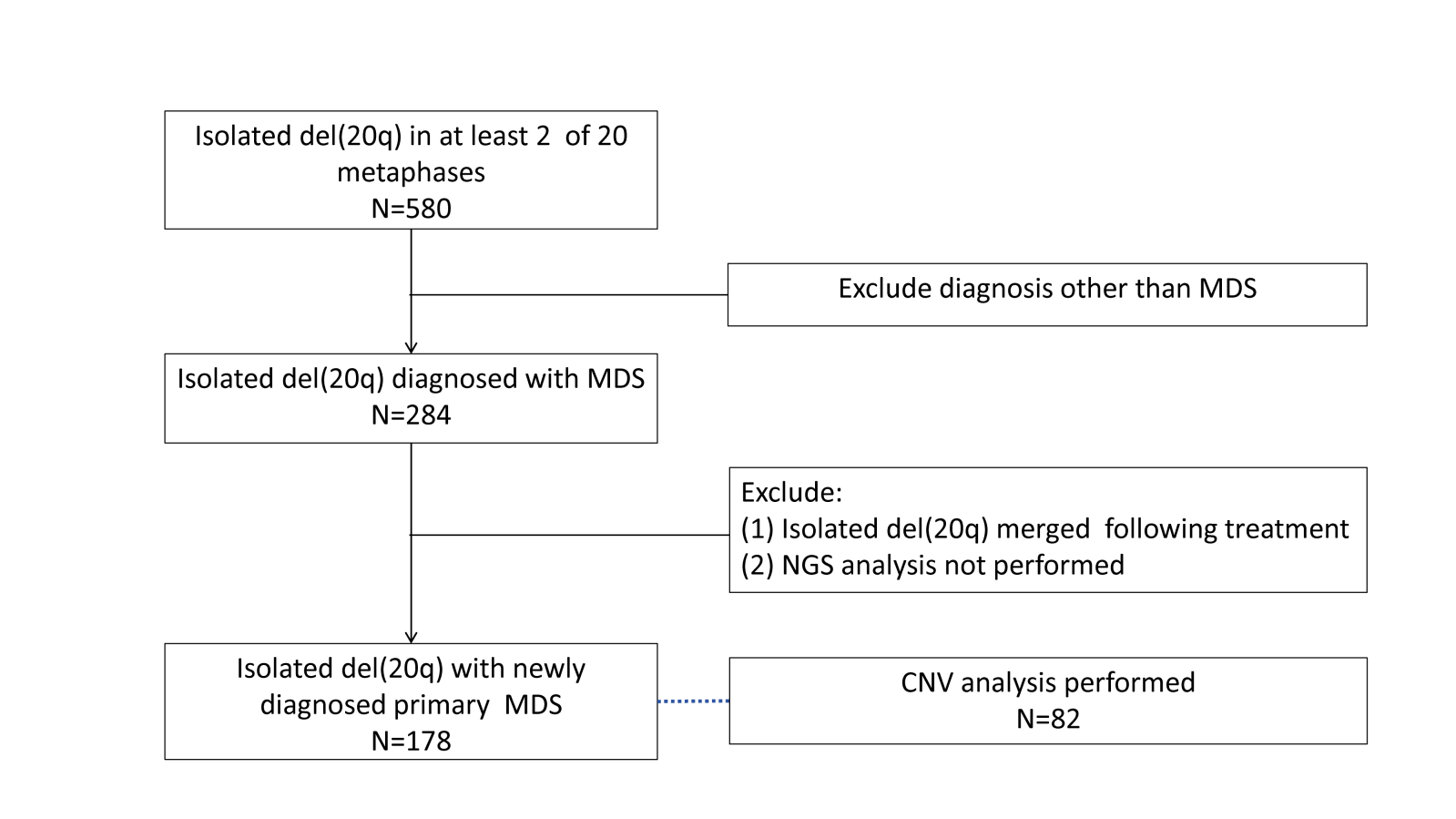
**

**Supplemental Figure 1. Flow diagram of patient cohort.**

178 patients were included in the cohort, for the CNV analysis 82 patients were included.

**
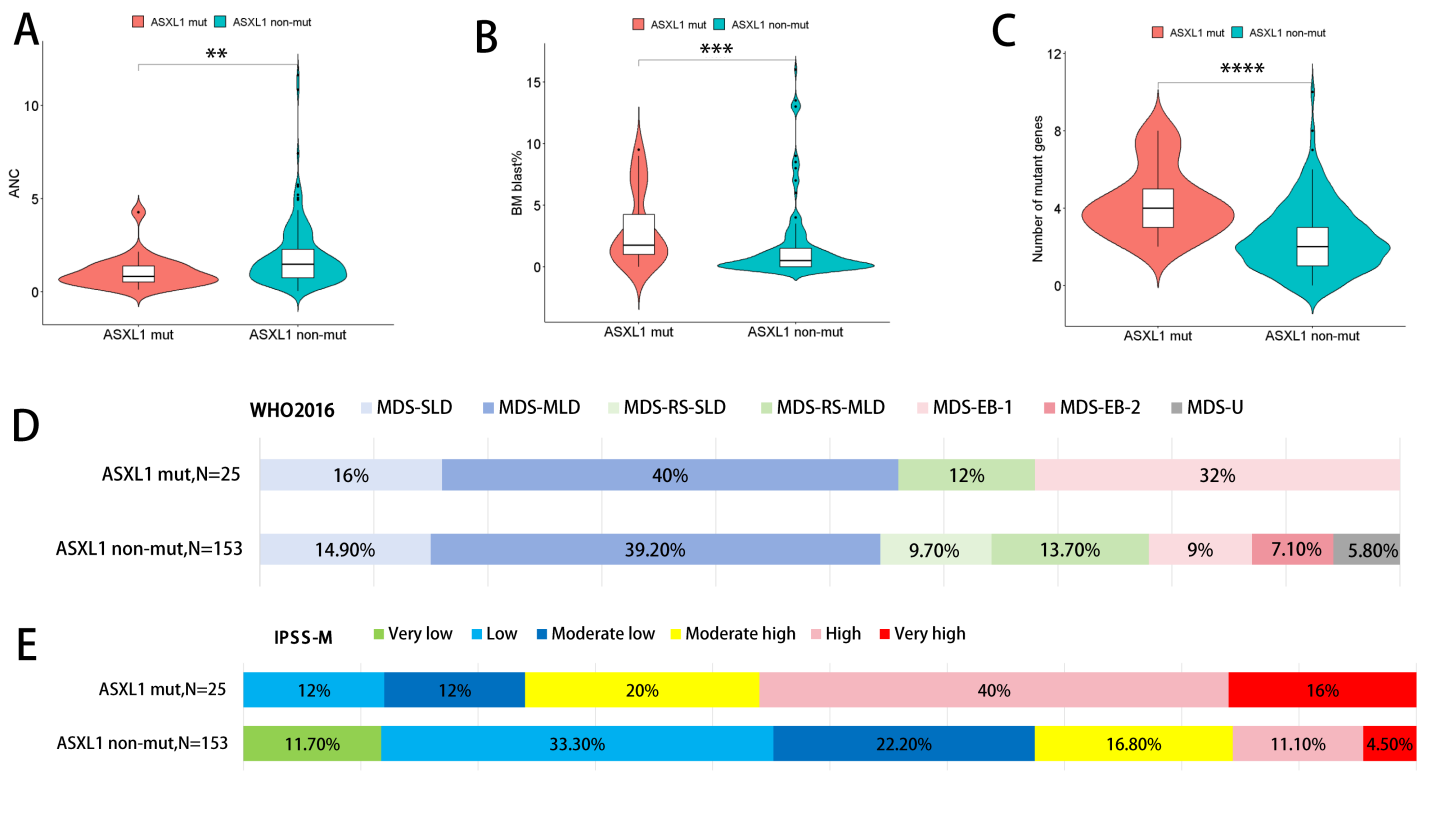
**

**Supplemental Figure 2. Clinical characteristics comparing cases in the *ASXL1* mutation group and *ASXL1* non-mutation group.**

Comparison of the distributions of ANC (x10^9^/L) (**A**), the proportion of bone marrow blasts (%) (**B**), and the number of mutant genes (**C**) between cases classified as *ASXL1*^mut^, *ASXL1*^non-mut^. *P*-values are derived from the Wilcoxon rank-sum test(A-C) . ***, *p*< 0.001; **, *p*<0.01. **D** Distribution of WHO (2016) classification in 178 MDS patients with isolated del(20q). The cohort was divided into two groups, those with *ASXL1* mutations and those without *ASXL1* mutations. **E** Distribution of IPSS-M risk in 178 MDS patients with isolated del(20q). The cohort was divided into two groups:those with *ASXL1* mutations and those without *ASXL1* mutations.

**
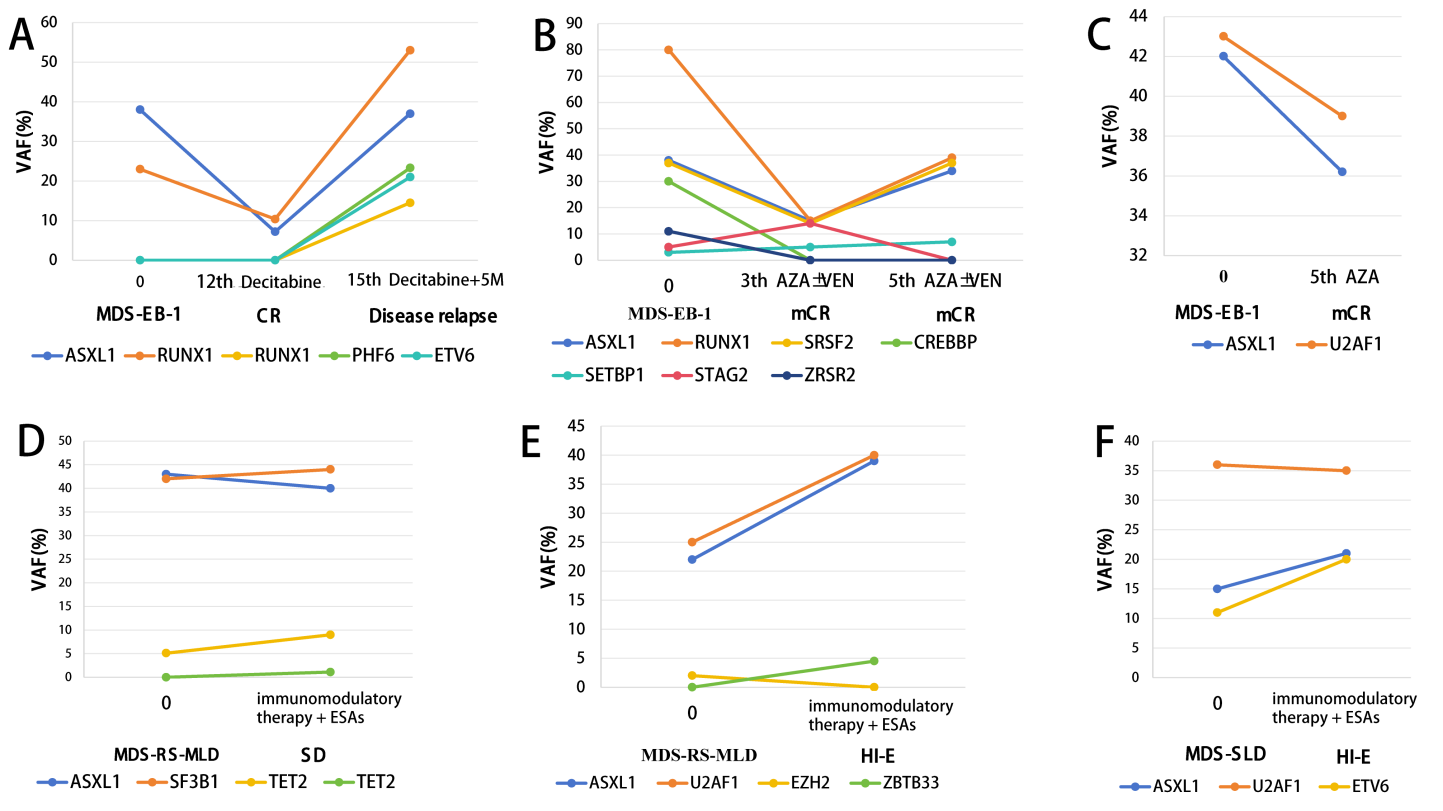
**

**Supplemental Figure 3. Clonal architecture of *ASXL1*-mutated MDS with isolated del(20q).**

The clonal dynamics of six patients, depicted using different line colors to represent the variant allele frequencies (VAF) of numerous mutations, are shown along with the acquisition of demethylating agents (**A–C**), supportive treatment (**D**), and immunosuppressive drugs (**E, F**).The VAF is plotted on the left y-axis across time points (x-axis). The initial time is defined as the time of diagnosis, marked as 0 months.

CR, complete remission; mCR, marrow complete remission; SD, stable disease; HI-E, hematologic improvement-erythroid; ESAs, erythropoiesis-stimulating agents; AZA, azacitidine; VEN, venetoclax

**Supplemental Table 1. The clinical information of MDS patients with isolated del(20q) and *ASXL1*^mut^ , *ASXL1* ^non-mut^ subtypes.**

| **Variables** | **Total** | ***ASXL1*** **^mut^** | ***ASXL1*** **^non-mut^** | ***P*** value |
| --- | --- | --- | --- | --- |
|  | **n=178** | **(n=25)^a^** | **(n=153)^a^** |  |
| **Male** | 111(62.4%) | 21(84%) | 90(58.8%) | **0.015^b^** |
| **Age at diagnosis** | 58(49,65) | 60(51,67.5) | 58(49,64) | 0.257^c^ |
| **Hemoglobin , g/L** | 84(66.75, 108) | 76(66.5,99) | 85(66.5,109) | 0.28^c^ |
| **ANC, ×10^9^**/L | 1.39(0.71, 2.23) | 0.8(0.5,1.51) | 1.5(0.7,2.3) | **0.006^c^** |
| **PLT, ×10^9^**/L | 61.5(31.75, 122) | 62(35,127) | 61(30.5,123) | 0.514^c^ |
| **BM Blasts (%)** | 0.5(0,2) | 1.5(1,5) | 0.5(0,1.5) | ＜**0.001^c^** |
| **Numbers of mutations** | 2 (1,4) | 4(3,5) | 2(1,3) | ＜0.001^c^ |
| **WHO(2016) classification** |  |  |  | **0.042^b^** |
| MDS-SLD | 27(15.1%) | 4(16%) | 23(14.9%) |  |
| MDS-MLD | 70(39.3%) | 10(40%) | 60(39.2%) |  |
| MDS-RS-SLD | 15(8.4%) | 0 | 15(9.7%) |  |
| MDS-RS-MLD | 24(13.4%) | 3(12%) | 21(13.7%) |  |
| MDS-EB-1 | 22(12.3%) | 8(32%) | 14(9%) |  |
| MDS-EB-2 | 11(6.1%) | 0 | 11(7.1%) |  |
| MDS-U | 9(5%) | 0 | 9(5.8%) |  |
| **IPSS-R risk group** |  |  |  | **0.038**^c^ |
| Very low | 19(10.6%) | 2(8%) | 17(11%) |  |
| low | 106(59.5%) | 11(44%) | 95(61.6%) |  |
| Intermediate | 38(21.3%) | 7(28%) | 32(20.7%) |  |
| High | 11(6.1%) | 5(20%) | 6(3.8%) |  |
| Very high | 4(2.2%) | 0 | 4(2.5%) |  |
| **IPSS-M risk group** |  |  |  | **0.001**^c^ |
| Very low | 18(10.1%) | 0 | 18(11.7%) |  |
| low | 54(30.3%) | 3(12%) | 51(33.3%) |  |
| Intermediate low | 37(20.7%) | 3(12%) | 34(22.2%) |  |
| Intermediate High | 31(17.4%) | 5(20%) | 26(16.8%) |  |
| High | 27(15.1%) | 10(40%) | 17(11.1%) |  |
| Very high | 11(6.1%) | 4(16%) | 7(4.5%) |  |

**ANC,absolute neutrophil count; PLT, platelet count;BM,bone marrow;WHO, World Health Organization; MDS-SLD,MDS with single lineage dysplasia; MDS-MLD, MDS with multilineage dysplasia;MDS-RS-SLD,MDS with ring sideroblasts (MDS-RS) with single lineage dysplasia ;MDS-RS-MLD,MDS-RS with multilineage; MDS-EB-1/2, MDS with excess blasts type 1/2; MDS-U, MDS unclassifiable; IPSS-R: Revised International Prognostic Scoring System；IPSS-M:International Prognostic Scoring System-Molecular.**

**Bold values: statistically significant p value.**

**a: Median (IQR); n (%).**

**b: Fisher’s exact test.**

**c: Mann-Whitney U test.**

**Supplemental Table 2. Gene list of the 267-gene NGS panel.**

| *ABCB1* | *ABL1* | *ANKRD26* | *APC* | *ARID1A* | *ARID1B* | *ARID2* | *ARID5B* | *ASXL1* | *ASXL2* |
| --- | --- | --- | --- | --- | --- | --- | --- | --- | --- |
| *ATG2B* | *ATM* | *ATRX* | *B2M* | *BACH2* | *BCL10* | *BCL2* | *BCL6* | *BCL7A* | *BCOR* |
| *BCORL1* | *BIRC3* | *BLM* | *BPGM* | *BRAF* | *BRCA1* | *BRCA2* | *BRIP1* | *BTG1* | *BTG2* |
| *BTK* | *CALR* | *CARD11* | *CBL* | *CBLB* | *CBLC* | *CCND1* | *CCND3* | *CCR4* | *CD28* |
| *CD58* | *CD79A* | *CD79B* | *CDC25C* | *CDKN1A* | *CDKN1B* | *CDKN2A* | *CDKN2B* | *CDKN2C* | *CEBPA* |
| *CHD2* | *CHD8* | *CIITA* | *CNOT3* | *CREBBP* | *CRLF2* | *CSF1R* | *CSF3R* | *CSMD1* | *CSNK1A1* |
| *CTCF* | *CUX1* | *CXCR4* | *CYLD* | *DDX3X* | *DDX41* | *DIS3* | *DKC1* | *DNM2* | *DNMT3A* |
| *DNMT3B* | *DTX1* | *DUSP2* | *EBF1* | *EED* | *EGFR* | *EGLN1* | *EGR1* | *ELANE* | *EP300* |
| *EPHA7* | *EPOR* | *ETNK1* | *ETV6* | *EZH2* | *FAM46C* | *FAS* | *FAT1* | *FAT4* | *FBXO11* |
| *FBXW7* | *FGFR3* | *FLT3* | *FOXO1* | *FYN* | *GAB2* | *GATA1* | *GATA2* | *GATA3* | *GFI1* |
| *GNA13* | *GNAI2* | *GNAS* | *GNB1* | *GSKIP* | *H1-2* | *H1-3* | *H1-4* | *H1-5* | *HAX1* |
| *HLA-A* | *HLA-C* | *HLA-DMB* | *HNRNPK* | *HRAS* | *HUWE1* | *HVCN1* | *ID3* | *IDH1* | *IDH2* |
| *IGLL5* | *IKZF1* | *IKZF2* | *IKZF3* | *IL7R* | *IRF2BP2* | *IRF4* | *IRF8* | *ITPKB* | *JAK1* |
| *JAK2* | *JAK3* | *JUNB* | *KDM6A* | *KIT* | *KLF2* | *KLHL6* | *KMT2A* | *KMT2B* | *KMT2C* |
| *KMT2D* | *KRAS* | *KRT20* | *LCOR* | *LMO2* | *LTB* | *LYN* | *MAP2K1* | *MAPK1* | *MAX* |
| *MCL1* | *MED12* | *MEF2B* | *MFHAS1* | *MPL* | *MTOR* | *MYC* | *MYCN* | *MYD88* | *MYOM2* |
| *NF1* | *NFE2* | *NFKBIA* | *NFKBIE* | *NOTCH1* | *NOTCH2* | *NOTCH3* | *NOTCH4* | *NPM1* | *NRAS* |
| *NT5C2* | *P2RY8* | *PALB2* | *PAX5* | *PDGFRA* | *PDGFRB* | *PDS5B* | *PHF6* | *PIGA* | *PIK3CA* |
| *PIK3CD* | *PIK3R1* | *PIM1* | *PIM2* | *PLCG1* | *PLCG2* | *POT1* | *PPM1D* | *PRDM1* | *PRF1* |
| *PRKCB* | *PRKD2* | *PRKDC* | *PRPF8* | *PRPS1* | *PSMB5* | *PTEN* | *PTPN1* | *PTPN11* | *PTPRD* |
| *RAD21* | *RASA2* | *RB1* | *RBBP6* | *RELN* | *RHOA* | *RPL10* | *RRAGC* | *RUNX1* | *SAMHD1* |
| *SBDS* | *SETBP1* | *SETD1B* | *SETD2* | *SETDB1* | *SF1* | *SF3B1* | *SGK1* | *SH2B3* | *SH2D1A* |
| *SMARCA4* | *SMC1A* | *SMC3* | *SMO* | *SOCS1* | *SP140* | *SPEN* | *SRP72* | *SRSF2* | *STAG2* |
| *STAT3* | *STAT5B* | *STAT6* | *SUFU* | *SUZ12* | *SYK* | *TAL1* | *TBL1XR1* | *TCF3* | *TERC* |
| *TERT* | *TET1* | *TET2* | *TMEM30A* | *TMSB4X* | *TNFAIP3* | *TNFRSF14* | *TOX* | *TP53* | *TPMT* |
| *TRAF3* | *U2AF1* | *UBE2A* | *UBR5* | *USP7* | *VAV1* | *VHL* | *WHSC1* | *WT1* | *XBP1* |
| *XPO1* | *ZAP70* | *ZBTB7A* | *ZFP36L1* | *ZMYM3* | *ZNF292* | *ZRSR2* |  |  |  |

**Supplemental Table 3. CNVs on chromosomes other than chromosome 20.**

| **Patient** | **WHO2016** | **Cytoband** | **Variant Type** | **Start** **position** | **End position** | **Ratio** | **Copy number** | **Gene related** |
| --- | --- | --- | --- | --- | --- | --- | --- | --- |
| #122 | MDS-SLD | 21q22.12 | deletion | 36164422 | 36421206 | 0.61 | 1.21 | RUNX1 |
| #117 | MDS-SLD | 12q24.11-q24.12 | deletion | 111087205 | 111886116 | 0.62 | 1.23 | SH2B3;HVCN1 |
| #126 | MDS-EB1 | 7q36.1 | duplication | 151833907 | 151877221 | 1.5 | 3.01 | KMT2C |
| #106 | MDS-EB2 | 11q23.3 | duplication | 118339490 | 118367082 | 1.76 | 3.51 | KMT2A |
|  |  | 12p13.2 | deletion | 12022358 | 12022903 | 0.52 | 1.04 | ETV6 |
|  |  | 13q13.1 | deletion | 32899213 | 32972704 | 0.73 | 1.46 | BRCA2 |
|  |  | 13q14.11 | deletion | 41133660 | 41240244 | 0.73 | 1.45 | FOXO1 |

**Supplemental Table 4. High-confidence mutations of 25 patients with ASXL1 variant.**

| **Patient** | **Gene** | **RefSeq** | **Position** | **Allele Change** | **Amino Acid Change** | **SNP_Accession** | **VAF(%)** |
| --- | --- | --- | --- | --- | --- | --- | --- |
| #11 | ASXL1 | NM_015338 | Exon 12 | c.2323delT | p.L775X | -- | 38.02 |
| #11 | EP300 | NM_001429 | Exon 6 | c.A1519G | p.S507G | rs146242251 | 51.83 |
| #11 | SH2B3 | NM_005475 | Exon 8 | c.G1606A | p.A536T | rs140649197 | 51.16 |
| #11 | RUNX1 | NM_001001890 | Exon 1 | c.A248G | p.K83R | -- | 23.18 |
| #3 | SETBP1 | NM_015559 | Exon4 | c.2602G>A | p.D868N | rs267607042 | 26.9 |
| #3 | U2AF1 | NM_001025203 | Exon2 | c.101C>T | p.S34F | rs371769427 | 42.4 |
| #3 | RELN | NM_005045 | Exon16 | c.1936C>T | p.R646W | rs115831287 | 46 |
| #3 | ASXL1 | NM_015338 | Exon12 | c.2324T>A | p.L775X | -- | 43.1 |
| #3 | TET2 | NM_001127208 | Exon3 | c.2440C>T | p.R814C | rs192553789 | 49 |
| #20 | NRAS | NM_002524 | Exon2 | c.35G>A | p.G12D | rs121913237 | 1.8 |
| #20 | U2AF1 | NM_001025203 | Exon2 | c.101C>T | p.S34F | rs371769427 | 38.7 |
| #20 | ASXL1 | NM_015338 | Exon12 | c.3083C>A | p.S1028X | rs200702600 | 1.4 |
| #20 | NOTCH1 | NM_017617 | Exon34 | c.6788G>A | p.R2263Q | rs200521815 | 33.2 |
| #21 | U2AF1 | NM_001025203 | Exon2 | c.101C>T | p.S34F | rs371769427 | 42.9 |
| #21 | ASXL1 | NM_015338 | Exon12 | c.2423delC | p.P808Lfs*10 | -- | 42.4 |
| #21 | CREBBP | NM_004380 | Exon31 | c.5234G>A | p.W1745X | -- | 45.1 |
| #21 | PLCG2 | NM_002661 | Exon14 | c.1343G>A | p.R448Q | -- | 52.2 |
| #21 | BCORL1 | NM_021946 | Exon7 | c.4171G>A | p.G1391R | rs199973665 | 98.9 |
| #21 | FAT1 | NM_005245 | Exon22 | c.11818A>G | p.T3940A | -- | 51.2 |
| #21 | FAT1 | NM_005245 | Exon10 | c.6265G>A | p.V2089I | rs76613365 | 51.4 |
| #41 | DNMT3A | NM_175629 | Exon23 | c.2645G>A | p.R882H | rs14700163 3 | 25.6 |
| #41 | PHF6 | NM_032458 | Exon8 | c.820C>T | p.R274* | -- | 30.6 |
| #41 | ASXL1 | NM_015338 | Exon12 | c.1900_192 2del23 | p.E635Rfs *15 | rs76643310 1 | 12.4 |
| #41 | GNB1 | NM_002074 | Exon5 | c.170A>T | p.K57M | rs74718351 7 | 2.1 |
| #52 | EZH2 | NM_004456 | Exon6 | c.619C>T | p.R207* | -- | 2 |
| #52 | U2AF1 | NM_006758 | exon2 | c.101C>T | p.S34F | rs37176942 7 | 25.3 |
| #52 | ASXL1 | NM_015338 | exon12 | c.1934dupG | p.G646Wfs *12 | rs75695815 9 | 21.6 |
| #212 | ASXL1 | NM_015338 | exon12 | c.2593G>T | p.E865* | -- | 47.1 |
| #212 | U2AF1 | NM_006758 | exon2 | c.101C>T | p.S34F | rs37176942 7 | 44 |
| #75 | JAK2 | NM_004972 | exon14 | c.1849G>T | p.V617F | rs77375493 | 2.2 |
| #75 | U2AF1 | NM_006758 | exon6 | c.470A>G | p.Q157R | rs371246226 | 2.1 |
| #75 | TET2 | NM_001127208 | exon3 | c.2355delT | p.H786Mfs*27 | -- | 2.3 |
| #75 | ASXL1 | NM_015338 | exon12 | c.2790_280 0dupGGAGAA AGCTG | p.A934Gfs *15 | -- | 1.8 |
| #76 | ASXL1 | NM_015338 | exon12 | c.2644C>T | p.Q882* | rs770209084 | 32.7 |
| #76 | U2AF1 | NM_006758 | exon2 | c.101C>T | p.S34F | rs37176942 7 | 38.7 |
| #76 | CSF3R | NM_156039 | exon17 | c.2053A>G | p.R685G | -- | 2.9 |
| #214 | ASXL1 | NM_015338 | exon13 | c.2269C>T | p.Q757* | rs77907882 6 | 22.8 |
| #214 | U2AF1 | NM_006758 | exon2 | c.101C>A | p.S34Y | rs37176942 7 | 23.6 |
| #96 | U2AF1 | NM_006758 | exon2 | c.101C>T | p.S34F | rs37176942 7 | 39.8 |
| #96 | ETV6 | NM_001987 | exon6 | c.1130C>T | p.A377V | -- | 14.6 |
| #96 | ASXL1 | NM_015338 | exon12 | c.1469T>C | p.I490T | -- | 2 |
| #210 | U2AF1 | NM_006758 | exon2 | c.101C>T | p.S34F | rs37176942 7 | 43.2 |
| #210 | TP53 | NM_000546 | exon7 | c.713G>A | p.C238Y | rs73088200 5 | 1.2 |
| #210 | ASXL1 | NM_015338 | exon12 | c.2036dupG | p.G680Rfs *38 | -- | 70.8 |
| #113 | ASXL1 | NM_015338 | exon13 | c.1927dupG | p.G646Wfs *11 | rs10853078 56 | 22.1 |
| #113 | U2AF1 | NM_006758 | exon2 | c.101C>T | p.S34F | rs37176942 7 | 36.7 |
| #113 | PRF1 | NM_001083116 | exon2 | c.445G>A | p.G149S | rs14746222 7 | 48.1 |
| #113 | DNMT3A | NM_175629 | intron17 | c.2083-2A>G | -- | rs761987159 | 3.6 |
| #173 | ETV6 | NM_001987 | exon7 | c.1195C>T | p.R399C | rs724159945 | 10.5 |
| #173 | U2AF1 | NM_006758 | exon2 | c.101C>T | p.S34F | rs371769427 | 35.9 |
| #173 | ASXL1 | NM_015338 | exon13 | c.2697dupA | p.P900Tfs*6 | -- | 14.5 |
| #127 | SF3B1 | NM_012433 | exon15 | c.2098A>G | p.K700E | rs559063155 | 42.3 |
| #127 | TET2 | NM_001127208 | exon3 | c.1630C>T | p.R544* | -- | 5.1 |
| #127 | TET2 | NM_001127208 | exon9 | c.4133G>A | p.C1378Y | rs765853421 | 47.3 |
| #127 | ASXL1 | NM_015338 | exon13 | c.1927dupG | p.G646Wfs*11 | rs1085307856 | 43.4 |
| #189 | ASXL1 | NM_015338 | exon12 | c.2077C>T | p.R693* | rs373221034 | 90.5 |
| #189 | U2AF1 | NM_006758 | exon2 | c.101C>A | p.S34Y | rs37176942 7 | 44.3 |
| #189 | GATA2 | NM_032638 | exon6 | c.1187G>A | p.R396Q | -- | 3.2 |
| #134 | TET2 | NM_001127208 | exon3 | c.1267A>T | p.K423* | -- | 90 |
| #134 | SRSF2 | NM_003016 | exon1 | c.284C>T | p.P95L | rs75171304 9 | 51.7 |
| #134 | ASXL1 | NM_015338 | exon12 | c.1934dupG | p.G646Wfs *12 | rs10853078 56 | 41.6 |
| #134 | STAG2 | NM_001042749 | exon20 | c.1840C>T | p.R614* | -- | 83.2 |
| #134 | STAG2 | NM_001042749 | exon12 | c.1038delA | p.K346Nfs *33 | -- | 2.3 |
| #134 | FAT4 | NM_024582 | exon2 | c.4027G>A | p.D1343N | -- | 3.1 |
| #145 | ASXL1 | NM_015338 | exon12 | c.1934dupG | p.G646Wfs*12 | rs1085307856 | 38 |
| #145 | SRSF2 | NM_003016 | exon1 | c.284C>G | p.P95R | rs751713049 | 38 |
| #145 | CREBBP | NM_004380 | exon30 | c.5138_5151del | p.H1713Lfs*17 |  | 30 |
| #145 | SETBP1 | NM_015559 | exon4 | c.2602G>A | p.D868N | rs267607042 | 2.9 |
| #145 | RUNX1 | NM_001754 | exon7 | c.710G>A | p.R237K | -- | 80 |
| #145 | NOTCH4 | NM_004557 | intron29 | c.5299-3C>G | -- | rs182937662 | 49.5 |
| #145 | PLCG2 | NM_002661 | intron23 | c.2514+2T>C | -- | -- | 49.8 |
| #145 | STAG2 | NM_001042749 | intron13 | c.1196+1G>T | -- | -- | 4.8 |
| #145 | ZRSR2 | NM_005089 | exon5 | c.377G>C | p.R126P | rs757655786 | 10.9 |
| #154 | ASXL1 | NM_015338 | exon12 | c.1934dupG | p.G646Wfs *12 | rs10853078 56 | 33.1 |
| #154 | U2AF1 | NM_006758 | exon2 | c.101C>T | p.S34F | rs37176942 7 | 33.4 |
| #154 | SETBP1 | NM_015559 | exon4 | c.2602G>A | p.D868N | rs26760704 2 | 1.3 |
| #154 | SETBP1 | NM_015559 | exon4 | c.2608G>A | p.G870S | rs26760704 0 | 2.9 |
| #154 | TERT | NM_198253 | exon11 | c.2830dupA | p.S944Kfs*95 | -- | 50.4 |
| #156 | ASXL1 | NM_015338 | exon12 | c.1934dupG | p.G646Wfs *12 | rs10853078 56 | 5.5 |
| #156 | U2AF1 | NM_006758 | exon2 | c.101C>T | p.S34F | rs37176942 7 | 27.2 |
| #156 | ZMYM3 | NM_201599 | exon15 | c.2428C>T | p.R810* | -- | 34 |
| #156 | ETV6 | NM_001987 | exon6 | c.1118C>T | p.P373L | -- | 5 |
| #160 | U2AF1 | NM_006758 | exon2 | c.101C>T | p.S34F | rs371769427 | 38.2 |
| #160 | ASXL1 | NM_015338 | exon12 | c.1934dupG | p.G646Wfs *12 | rs1085307856 | 36.1 |
| #161 | U2AF1 | NM_006758 | exon2 | c.101C>A | p.S34Y | rs371769427 | 47.2 |
| #161 | CREBBP | NM_004380 | exon30 | c.5167_516 8insCACTGT GT | p.C1723Sfs*24 | -- | 31.9 |
| #161 | ASXL1 | NM_015338 | exon12 | c.2411_241 2insACCC | p.V807Hfs*16 | -- | 32 |
| #161 | RUNX1 | NM_001754 | exon6 | c.601C>T | p.R201* | rs1057519748 | 27.8 |
| #161 | CBL | NM_005188 | exon8 | c.1150T>C | p.C384R | rs387906664 | 2.3 |
| #161 | SETD1B | NM_001353345 | exon13 | c.5240_525 8del | p.R1748Pfs*28 | -- | 3.7 |
| #161 | TET2 | NM_001127208 | exon4 | c.3452T>G | p.L1151R | -- | 11.5 |
| #213 | DNMT3A | NM_022552 | exon18 | c.2093G>A | p.W698* | rs10053597 74 | 41.1 |
| #213 | SRSF2 | NM_003016 | exon1 | c.284C>A | p.P95H | rs75171304 9 | 42.1 |
| #213 | SETBP1 | NM_015559 | exon4 | c.2602G>T | p.D868Y | -- | 42.6 |
| #213 | ASXL1 | NM_015338 | exon13 | c.1934dupG | p.G646Wfs *12 | rs10853078 56 | 36.7 |
| #213 | ATR | NM_001184 | exon8 | c.1819_182 1delinsA | p.C607Ifs *9 | -- | 45.2 |
| #187 | U2AF1 | NM_001025203 | Exon2 | c.101C>T | p.S34F | rs371769427 | 31.5 |
| #187 | KMT2D | NM_003482 | Exon6 | c.688G>A | p.A230T | -- | 49.8 |
| #187 | MPL | NM_005373 | Exon11 | c.1652C>T | p.P551L | -- | 47.2 |
| #187 | EP300 | NM_001429 | Exon12 | c.2240C>T | p.P747L | rs193026103 | 48.3 |
| #187 | PHF6 | NM_032458 | Exon9 | c.872G>A | p.G291E | -- | 12.1 |
| #187 | BCORL1 | NM_021946 | Exon7 | c.4171G>A | p.G1391R | rs199973665 | 100 |
| #187 | ASXL1 | NM_015338 | Exon12 | c.3019_3020insTTCA | p.G1007Vfs*10 | -- | 33.5 |
| #187 | CREBBP | NM_004380 | Exon7 | c.1651C>A | p.L551I | rs61753381 | 47.9 |
| #188 | U2AF1 | NM_001025203 | Exon6 | c.470A>G | p.Q157R | rs371246226 |  |
| #188 | NOTCH1 | NM_017617 | Exon7 | c.1214C>T | p.T405M | rs370746889 |  |
| #188 | ASXL1 | NM_015338 | Exon11 | c.1347C>A | p.Y449X | -- |  |

**Supplemental Table 5. The clinical information of lower-risk( IPSS-R score≤3.5 ) MDS patients with isolated del(20q) and classified as *ASXL1* ^mut^,*ASXL1* ^non-mut^ subtypes.**

| **Variables** | **Total** | **ASXL1 ^mut^** | **ASXL1 ^non-mut^** | ***P* value** |
| --- | --- | --- | --- | --- |
|  | **(n=142)^a^** | **(n=18)^a^** | **(n=124)^a^** |  |
| **Male** | 86(60.6%) | 14(77.8%) | 72(58.1%) | 0.128^b^ |
| **Age at diagnosis,y** | 58(48.8,64) | 59.5(50.3,66.3) | 57(48.3,63.8) | 0.331^c^ |
| **Hemoglobin, g/L** | 88.5(71,109.3) | 81.5(68.3,106.3) | 90.5(71,109.8) | 0.56^c^ |
| **ANC, ×10^9^/L** | 1.5(0.9,2.3) | 1.2(0.7,1.6) | 1.6(1,2.3) | 0.06^c^ |
| **PLT, ×10^9^/L** | 67.5(35,133) | 89(39.8,147.8) | 66(35,130.8) | 0.28^c^ |
| **BM Blasts,%** | 0.5(0,1) | 1(0.5,2.3) | 0.5(0,1) | **0.002**^c^ |
| **Numbers of mutations** | 2 (1,4) | 4(3,5) | 2(1,3) | **＜0.001**^c^ |
| **WHO(2016) classification** |  |  |  | **0.037^b^** |
| MDS-SLD | 26(18.3%) | 4(22.2%) | 22(17.7%) |  |
| MDS-MLD | 65(45.7%) | 8(44.4%) | 54(43.5%) |  |
| MDS-RS-SLD | 15(10.5%) | 0 | 15(12%) |  |
| MDS-RS-MLD | 20(14%) | 3(16.6%) | 20(16.1%) |  |
| MDS-EB-1 | 4(2.8%) | 3(16.6%) | 1(0.8%) |  |
| MDS-EB-2 | 3(2.1%) | 0 | 3(2.4%) |  |
| MDS-U | 9(6.3%) | 0 | 9(7.2%) |  |
| **IPSS-R risk group** |  |  |  | 0.11^c^ |
| Very low | 19(13.4%) | 2(11.1%) | 17(13.7%) |  |
| low | 106(74.6%) | 11(61.1%) | 95(76.6%) |  |
| Intermediate | 17(12%) | 5(27.7%) | 12(9.6%) |  |
| High | 0 | 0 | 0 |  |
| Very high | 0 | 0 | 0 |  |
| **IPSS-M risk group** |  |  |  | **＜0.001**^c^ |
| Very low | 18(12.6%) | 0 | 18(14.5%) |  |
| low | 53(37.3%) | 3(16.6%) | 50(40.3%) |  |
| Intermediate low | 33(23.2%) | 3(16.6%) | 30(24.1%) |  |
| Intermediate High | 22(16.1%) | 5(27.7%) | 18(14.5%) |  |
| High | 11(7.7%) | 5(27.7%) | 6(4.8%) |  |
| Very high | 4(2.8%) | 2(11.1%) | 2(1.6%) |  |

**Bold values: statistically significant p value.**

**a: Median (IQR); n (%).**

**b: Fisher’s exact test.**

**c: Mann-Whitney U test.**

**Supplemental Table 6. The clinical information of higher-risk ( IPSS-R score＞3.5 ) MDS patients with isolated del(20q) and classified as *ASXL1* ^mut^ subtype, *ASXL1* ^non-mut^ subtype.**

| **Variables** | **Total** | **ASXL1 ^mut^** | **ASXL1 ^non-mut^** | ***P* value** |
| --- | --- | --- | --- | --- |
|  | **(n=36)^a^** | **(n=7)^a^** | **(n=29)^a^** |  |
| **Male** | 25(69.4%) | 7(100%) | 18(62.1%) | 0.076^b^ |
| **Age at diagnosis,y** | 60(49.5,68.5) | 60(52,70) | 59(49,68) | 0.734^c^ |
| **Hemoglobin, g/L** | 69.5(56.3,86.5) | 69(52,81) | 70(56.5,88) | 0.589^c^ |
| **ANC, ×10^9^/L** | 0.6(0.4,1.3) | 0.4(0.4,0.8) | 0.6(0.5,2) | 0.156^c^ |
| **PLT, ×10^9^/L** | 36.5(20.3,60.5) | 40(25,47) | 33(20,61.5) | 0.719^c^ |
| **BM Blasts,%** | 6.3(2.5,8.9) | 6.5(2.5,9) | 6(2.5,8.8) | 0.936^c^ |
| **Numbers of mutations** | 3 (2,4) | 4(3,6) | 2(2,3) | **0.011**^c^ |
| **WHO(2016) classification** |  |  |  | 0.606^b^ |
| MDS-SLD | 1(2.7%) | 0 | 1(3.4%) |  |
| MDS-MLD | 8(22.2%) | 2(28.5%) | 6(20.6%) |  |
| MDS-RS-SLD | 0 | 0 | 0 |  |
| MDS-RS-MLD | 1(2.7%) | 0 | 1(3.4%) |  |
| MDS-EB-1 | 18(50%) | 5(71.5%) | 13(44.8%) |  |
| MDS-EB-2 | 8(22.2%) | 0 | 8(27.5%) |  |
| MDS-U | 0 | 0 | 0 |  |
| **IPSS-R risk group** |  |  |  | 0.29^c^ |
| Very low | 0 | 0 | 0 |  |
| low | 0 | 0 | 0 |  |
| Intermediate | 21(58.3%) | 2(28.5%) | 19(65.5%) |  |
| High | 11(30.5%) | 5(71.5%) | 6(20.6%) |  |
| Very high | 4(11.1%) | 0 | 4(13.7%) |  |
| **IPSS-M risk group** |  |  |  | 0.078^c^ |
| Very low | 0 | 0 | 0 |  |
| low | 1(2.7%) | 0 | 1(3.4%) |  |
| Intermediate low | 4(11.1%) | 0 | 4(13.7%) |  |
| Intermediate High | 8(22.2%) | 0 | 8(27.5%) |  |
| High | 16(44.4%) | 5(71.5%) | 11(37.9%) |  |
| Very high | 7(19.4%) | 2(28.5%) | 5(17.2%) |  |

**Bold values: statistically significant p value.**

**a: Median (IQR); n (%).**

**b: Fisher’s exact test.**

**c: Mann-Whitney U test.**

**Supplemental Table 7. The clinical information of MDS patients with isolated del(20q) and classified as *ASXL1* ^only-mut^ , *ASXL1* ^only-del^ , *ASXL1* ^wt^ subtypes.**

| **Variables** | ***ASXL1*^only-del^** | ***ASXL1*^only-mut^** | ***ASXL1* ^wt^** | ***P* value** | **Del vs. Wt** | **Mut vs. Wt** | **Del vs. Mut** |
| --- | --- | --- | --- | --- | --- | --- | --- |
|  | **(n=20)^a^** | **(n=9)^a^** | **(n=51)^a^** |  |  |  |  |
| **Male** | 11(55%) | 7(77.8%) | 29(56.9%) | 0.49**^b^** | - | - | - |
| **Age at diagnosis,y** | 50(47,64) | 61(49.5,71) | 59(55,65) | 0.11^c^ | - | - | - |
| **Hemoglobin, g/L** | 103.5(80.5,119) | 71(59.5,99) | 74(62,95) | **0.012**^c^ | **0.004^d^** | 0.844**^d^** | **0.036^d^** |
| **ANC, ×10^9^/L** | 2.6(1.1,3.6) | 0.8(0.7,1.6) | 1.3(0.7,1.9) | **0.013**^c^ | **0.006^d^** | 0.475**^d^** | **0.03^d^** |
| **PLT, ×10^9^/L** | 33(13.5,70.8) | 93(44.5,196.5) | 66(39,136) | **0.002**^c^ | **0.002^d^** | 0.362**^d^** | **0.008^d^** |
| **BM Blasts,%** | 1(0.1,1.5) | 1(0.5,5.3) | 1(0,3) | 0.66^c^ | - | - | - |
| **Numbers of mutations** | 2(1,3.8) | 4(3,6) | 2(2,3) | **0.007**^c^ | 0.922**^d^** | **0.001^d^** | **0.017^d^** |
| **WHO(2016) classification** |  |  |  | 0.24**^b^** | - | - | - |
| MDS-SLD | 8(40%) | 2(22.2%) | 6(11.7%) |  |  |  |  |
| MDS-MLD | 7(35%) | 3(33.3%) | 19(37.2%) |  |  |  |  |
| MDS-RS-SLD | 2(10%) | 0 | 6(11.7%) |  |  |  |  |
| MDS-RS-MLD | 0 | 2(22.2%) | 7(13.7%) |  |  |  |  |
| MDS-EB-1 | 1(5%) | 2(22.2%) | 9(17.6%) |  |  |  |  |
| MDS-EB-2 | 1(5%) | 0 | 3(5.8%) |  |  |  |  |
| MDS-U | 1(5%) | 0 | 1(1.9%) |  |  |  |  |
| **IPSS-R risk group** |  |  |  | 0.20^c^ | - | - | - |
| Very low | 3(15%) | 0 | 5(9.8%) |  |  |  |  |
| low | 14(70%) | 5(55.5%) | 28(54.9%) |  |  |  |  |
| Intermediate | 1(5%) | 3(33.3%) | 14(27.4%) |  |  |  |  |
| High | 1(5%) | 1(11.1%) | 3(5.8%) |  |  |  |  |
| Very high | 1(5%) | 0 | 1(1.9%) |  |  |  |  |
| **IPSS-M risk group** |  |  |  | **0.001^c^** | **0.032^d^** | **0.004^d^** | **0.002^d^** |
| Very low | 3(15%) | 0 | 3(5.8%) |  |  |  |  |
| low | 10(50%) | 0 | 11(21.5%) |  |  |  |  |
| Intermediate low | 2(10%) | 1(11.1%) | 16(31.3%) |  |  |  |  |
| Intermediate High | 2(10%) | 2(22.2%) | 10(19.6%) |  |  |  |  |
| High | 1(5%) | 4(44.4%) | 8(15.6%) |  |  |  |  |
| Very high | 2(10%) | 2(22.2%) | 3(5.8%) |  |  |  |  |

**Bold values: statistically significant p value.**

1. **Median (IQR); n (%).**
2. **Fisher’s exact test.**
3. **Kruskal-Wallis H test.**
4. **Mann-Whitney U test.**
